# Impact of coronavirus disease 2019 on chronic pain structures: data from French national survey

**DOI:** 10.2217/cer-2022-0003

**Published:** 2022-05-05

**Authors:** Meggane Melchior, Mikhail Dziadzko, Séverine Conradi, Pierrick Poisbeau, Frédéric Aubrun

**Affiliations:** ^1^Institut des Neurosciences Cellulaires et Intégratives, Centre National de la Recherche Scientifique et Université de Strasbourg, Strasbourg, 67000, France; ^2^Département d'Anesthésie-Réanimation-Douleur, Hôpital de la Croix-Rousse, Hospices Civils de Lyon, Université Claude Bernard Lyon 1, Lyon, 69004, France; ^3^Research on Healthcare Performance, Institut National de la Santé et de la Recherche Médicale U1290, Université Claude Bernard Lyon 1, Lyon, 69373, France; ^4^Centre Hospitalier Régional Universitaire de Tours de Nancy – Hôpital Central, Nancy, 54000, France; ^5^Laboratoire APEMAC-EPSAM Université de Lorraine, Metz, 57000, France

**Keywords:** chronic pain structure, COVID-19, human resource allocation, pain management, pain specialists

## Abstract

**Aims:** The authors evaluated the impact of the first coronavirus disease 2019 pandemic wave on French chronic pain structures (CPSs). **Methods:** An online survey assessed CPS resource allocation, workflow and perceived impact on patient care. **Results:** All CPS workflow was severely impacted by the reallocation of 42% of specialists. In-person appointments were cancelled by 72% of participants. Follow-up was maintained in 91% of participants (telemedicine). Skills in end-of-life decision-making/counseling were rarely solicited. The perceived impact of the crisis on the experience of patients was high (eight out of ten), with a significant increase in access-to-care delay. **Conclusion:** CPSs maintained patient follow-up. Special features of CPS specialists were rarely solicited by coronavirus disease 2019 teams experiencing a high workload. Recommendations on optimal CPS resource reallocations have to be standardized in crisis conditions.

The ongoing coronavirus disease 2019 (COVID-19) pandemic declared by the WHO in March 2020 has profoundly affected the healthcare system. Strict country-specific measures have been taken all over the world to fight the crisis and to manage the surge of COVID-19 patients. In March 2020, the French government declared stage 3 of the Organisation de la Réponse du Système de Santé en Situation Sanitaire Exceptionnelle emergency preparedness plan to set up a specific organization for healthcare structures (‘Plan Blanc’) [1]. ‘Plan Blanc' defines the operational conditions of healthcare facilities and allows the allocation of healthcare human resources according to sanitary situations [2]. This health state of emergency deployment has led to the cancellation of most nonurgent procedures and appointments. Healthcare human resources, including medical staff, were reallocated to COVID-19 centers to reinforce hospital needs in the face of the high number of patients with SARS coronavirus 2 (SARS-CoV-2). These measures had a strong impact on routine healthcare activities and the follow-up of patients with chronic diseases, such as chronic pain. In several countries, patients with chronic pain were not able to continue the usual follow-up [3–6]. This has been associated with changes in pain treatment and modifications in psychological support [7], leading to exacerbation of pain symptoms [8–13]. Telehealth solutions, which were developed before the pandemic, were suggested [14].

The reported prevalence of chronic pain in the French population is about 30% (approximately 22 million people), and 5% of the adult population present moderate to severe chronic pain with neuropathic characteristics [15]. Such conditions lead to disability, strongly affecting quality of life, often needing a medicalized approach and resulting in an economic burden [16–18]. Recent evidence on the persistent pain syndrome in COVID-19 survivors may definitely increase the disease burden in the population [19,20].

French chronic pain structures (CPSs), created in the early 2000s, offers multidisciplinary pain care. More than 240 French CPSs (including 38 pediatric structures) were functional before the COVID-19 outbreak, with an annual estimated volume of 400,000 patients [21]. The typical CPS team is pluriprofessional and is composed of at least 0.5 full-time equivalent physicians and 1.5 full-time equivalent paramedical staff, including nurses, physical therapists, psychologists and administrative personnel [22]. Most CPSs manage all types of chronic pain patients. Only a minority of CPSs are specialized in one specific type of patient (e.g., cancer or pediatric patients). A small proportion of CPSs offer palliative care. In the majority of hospitals, palliative care is an independently functioning unit; however, CPS staff may provide *ad hoc* care. Most French CPS centers did not use telemedicine before the pandemic crisis, but a telephone follow-up was used frequently.

Following the implementation of ‘Plan Blanc', a large proportion of pain physicians and paramedical staff were allocated to different hospital units. National directives encouraged physical distancing and online medical consultations. As such, French CPSs have had to adapt their workflow, mostly by limiting in-person consultations, suspending or delaying pain management procedures and using telemedicine [8,23,24].

The objective of this study was to assess CPS human resource allocation, changes in CPS workflow organization and the perceived impact on patient care in the context of the COVID-19 pandemic sanitary crisis. The data obtained will allow us to discuss the workload of French pain specialists, initiate strategies to prevent psychological impact on caregivers and develop possible strategies to maintain patient access to care during similar crises and beyond.

## Methods

A semistructured mixed qualitative/quantitative survey research design was used with nonprobability sampling (convenience sample). For the purpose of the study, an anonymous French language online survey, including multiple choice and open questions, was developed by the board of the Société Française d'Etude et de Traitement de la Douleur (French Society of Pain Management). The Société Française d'Etude et de Traitement de la Douleur is the French chapter of the International Association for the Study of Pain and a member of the European Pain Federation. The computer-administered survey aimed to identify the new missions given to CPS human resources, assess the strategies to maintain follow-up of chronic pain patients, evaluate the overall impact on the functioning of CPSs, evaluate the perceived impact on patient care and assess whether there were any supply/medication issues or shortages during the period of ‘Plan Blanc' in the region of the participant's practice.

A Delphi method was used to design the survey. After identification of the participant's role and affiliation (region, city, profession), the survey comprised of 57 items divided into three categories: current professional appointment (reallocation, same position as before the crisis, etc.) and functions, resources and solutions to maintain chronic pain patient follow-up and information on difficulties with the supply of medications and medical devices. Response options were binary (yes/no), categorical (preselected lists of items), 11-item scales (0 = least important; 10 = most important), continuous (mostly for time-related questions) and open text. The translated questionnaire is provided in the Supplementary data.

The survey invitation was distributed by email to all members of the Société Française d'Etude et de Traitement de la Douleur working in French CPSs 1 month after the ‘Plan Blanc' termination beginning in June 2020 and made available up to August 2020, with three reminders. Participants were not limited to physicians; all members of CPSs were welcome to participate. Survey participation was voluntary, and no compensation was offered. A secure web application was used for building, collecting and managing the survey data. This study has been declared to the Commission Nationale de L'informatique et des Libertés (French Data Protection Agency).

### Statistical analysis

Most data were analyzed descriptively and presented as frequency and percentage or as median and interquartile range (IQR) accordingly. Some participants did not answer all questions or provided answers that could not be analyzed. Percentages were calculated based on the number of responses to each question. Open questions were manually reviewed to identify keywords, and were ‘pile-sorted’ and reported as frequency and percentage whenever possible. Data comparison was made using the Wilcoxon matched-pairs signed rank test or chi-square test. A p < 0.05 was considered statistically significant.

## Results

### Participants

In total, 224 responses were collected. Most participants were physicians (65%; n = 145), followed by nurses (22%; n = 49), psychologists (9%; n = 21), secretaries (1%; n = 2) and others (3%; n = 7). Most participants were from the mainland.

### Human resource reallocation

**Figure 1.**
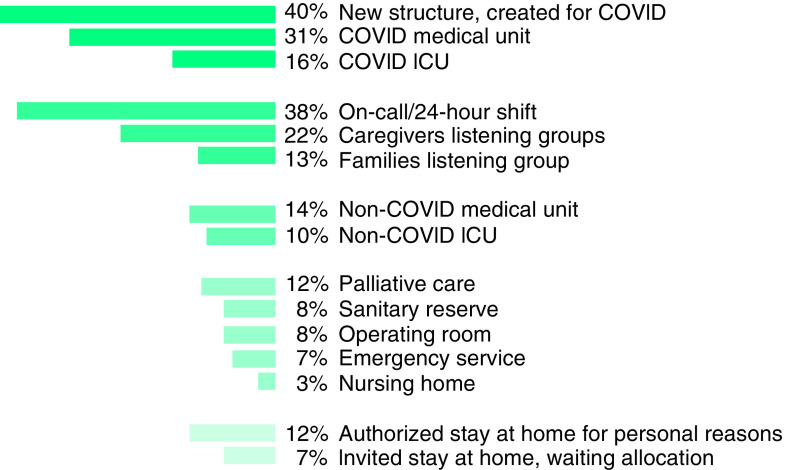
Participant allocations during the coronavirus disease crisis. The percentage of participants responding yes to the different items is presented. ICU: Intensive care unit.

Of the 224 respondents, 93 (42%) were reallocated to different medical services and were not able to maintain their primary activity at the CPS. Among all respondents, 143 (64%) were volunteers to be reallocated. Half (72 of 143) were indeed reallocated, and 28% (23 of 81) of the participants who were not volunteers were nevertheless reallocated, responding to institutional and sanitary needs. More than a third of participants were assigned to COVID-19 patient care units, including medicine and intensive care units (ICUs) (Figure 1 & Table 1).

**Table 1.** Chronic pain structure human resource allocations during the coronavirus disease 2019 crisis

All respondents	Overall n = 224	Physicians n = 145 (64%)	Nurses n = 49 (23%)	Psychologists n = 21 (9%)	Secretaries n = 2 (1%)	Other n = 7 (3%)
Stayed at their position	130 (58%)	92 (67%)	14 (29%)	14 (67%)	2 (100%)	3 (43%)
Volunteered to be reallocated	143 (64%)	92 (63%)	35 (71%)	10 (48%)	1 (50%)	4 (57%)

Areas of reallocation^†^						
COVID medical unit	69 (31%)	46 (32%)	15 (31%)	7 (33)	0 (0%)	1 (14)
COVID ICU	36 (16%)	21 (15%)	12 (24%)	3 (14)	0 (0%)	0 (0%)
Operating room	17 (8%)	12 (8%)	5 (10%)	0 (0%)	0 (0%)	0 (0%)
Non-COVID ICU	22 (10%)	15 (10%)	5 (10%)	1 (5%)	0 (0%)	1 (14%)
Nursing home	7 (3%)	5 (3%)	1 (2%)	1 (5%)	0 (0%)	0 (0%)
Palliative care	27 (12%)	20 (14%)	5 (10%)	1 (5%)	1 (50%)	0 (0%)
Non-COVID medical/surgical unit	32 (14%)	19 (13%)	11 (22%)	2 (10%)	0 (0%)	0 (0%)
Sanitary reserve	17 (8%)	12 (8%)	3 (6%)	2 (10%)	0 (0%)	0 (0%)
SAMU/SMUR emergency medical services	15 (7%)	12 (8%)	2 (4%)	1 (5%)	0 (0%)	0 (0%)
Family listening group	29 (13%)	15 (10%)	3 (6%)	10 (48%)	1 (50%)	0 (0%)
Caregiver listening group	49 (22%)	25 (17%)	4 (8%)	17 (81%)	1 (50%)	2 (29%)
Invited to stay home while awaiting an allocation	15 (7%)	10 (7%)	1 (2%)	4 (19%)	0 (0%)	0 (0%)
Authorized to stay home for personal reasons	26 (12%)	17 (12%)	4 (8%)	4 (19%)	1 (50%)	0 (0%)
On-call or 24-h shift care	86 (38%)	69 (48%)	10 (20%)	3 (14%)	1 (50%)	4 (43)

Detailed activity for those who were reallocated to a new structure created for COVID^‡^	Overall n = 89 (40%)	Physicians n = 59 (41%)	Nurses n = 17 (35%)	Psychologists n = 6 (29%)	Secretaries n = 2 (100%)	Other n = 4 (57%)
Chronic pain teleconsultation	8 (9%)	7 (12%)	1 (6%)	0 (0%)	0 (0%)	0 (0%)
Management and ethics COVID crisis cells	6 (7%)	5 (8%)	0 (0%)	0 (0%)	0 (0%)	1 (25%)
COVID teleconsultation	5 (6%)	3 (5%)	2 (12%)	0 (0%)	0 (0%)	0 (0%)
COVID unit	7 (8%)	5 (8%)	2 (12%)	0 (0%)	0 (0%)	0 (0%)
Education	1 (1%)	0 (0%)	0 (0%)	0 (0%)	0 (0%)	1 (25%)
Family/patient psychological hotline	9 (10%)	6 (10%)	1 (6%)	1 (17%)	1 (50%)	0 (0%)
ICU/emergency room night shift	4 (5%)	1 (2%)	0 (0%)	3 (50%)	0 (0%)	0 (0%)
Non-COVID unit	10 (11%)	10 (17%)	0 (0%)	0 (0%)	0 (0%)	0 (0%)
On call for chronic pain and palliative care	4 (5%)	4 (7%)	0 (0%)	0 (0%)	0 (0%)	0 (0%)
Psychological/medical support for clinicians	12 (14%)	10 (17%)	2 (12%)	0 (0%)	0 (0%)	0 (0%)
Respiratory tract/serum sampling for COVID	13 (15%)	7 (12%)	1 (6%)	2 (33%)	1 (50%)	2 (50%)

† Most reallocated respondents combined different activities (i.e., answered yes for multiple items) or participated in a COVID-related unit on top of their initial position (i.e., answered yes to ‘could you stay at your initial position’ and multiple other items).

‡ Some participants did not answer all questions or provided answers that could not be analyzed. Percentages were calculated based on the number of responses to each question.

COVID: Coronavirus disease; ICU: Intensive care unit; SAMU/SMUR: Service d'aide médicale urgente/services mobiles d'urgence et de réanimation.

Almost half (48%; n = 69) of the physicians participated in on-call or at-hospital 24-h shift care. 76 (58%) of the 130 participants who could stay at their initial position also worked in a COVID or non-COVID ICU, operating room, nursing home, palliative care, non-COVID medical or surgical unit, sanitary reserve, emergency medical services or on-call or 24-h shift care. A heterogeneous proportion of respondents participated in psychological support offers (listening groups). There were 29 (13%) different specialists in listening groups for patients and families - including psychologists (48%; n = 10), physicians (10%; n = 15), and nurses (6%; n = 3). 49 (22%) specialists were offering psychological support for clinicians. The following CPS specialists were involved – psychologists (81%; n = 17); physicians (17%; n = 25); nurses (8%; n = 4). Reallocated respondents often combined different activities. 89 respondents detailed their functions in newly created structures. Physicians (n = 59) mainly joined chronic pain telemedicine consultations, COVID crisis cells (management and ethics), COVID units or consultations, ICU or emergency room night shifts, non-COVID units or on-call chronic pain and palliative care. Pain nurses (n = 17) mainly performed biological samplings for COVID patients. Psychologists (n = 6) were mainly involved in the creation of a family/patient psychological hotline as well as psychological or medical support for clinicians. Table 1 details the different areas in which participants were allocated.

### Maintaining in-person appointments & the use of telemedicine

A total of 28% (55 of 197) of respondents maintained in-person appointments, mostly for urgent reasons, including patients with severe pain, acute-on-chronic pain, acute neuropathic or facial pain (36%; n = 20), cancer or palliative care (33%; n = 18) or interventional pain treatment procedures (5%; n = 3), and for new patients (9%; n = 5). One respondent maintained in-person appointments for clinicians/colleagues. 11 (20%) participants did not specify their criteria for in-person appointments.

Telemedicine solutions, including telephone calls and video consultations, were used by 91% (180 of 197) of respondents. Telephone calls were used by 92% (n = 165) of telemedicine users, and 49% (n = 89) used video consultation through online software. Video calls were used in more than half (64%; n = 116) of cases involving existing patients but also for new patients (29%; n = 52), patients who specifically asked for them (48%; n = 87) and patients with specific criteria (36%; n = 65). These criteria included unsatisfactory pain management/re-evaluation of treatment (17%; n = 11), anxiety or emotional troubles (15%; n = 10), transcutaneous electrical nerve stimulation (TENS) follow-up (9%; n = 6), cancer or chemotherapy (9%; n = 6), acute pain or reactivation of symptoms (5%; n = 3) and risk of severe COVID (5%; n = 3). 15 (23%) participants did not specify their criteria.

Among respondents who were not familiar with telemedicine consultations before the COVID-19 crisis, 57% (85 of 150) recognized telephone consultations as useful and 40% (n = 60) considered them a precious tool. Video-assisted consultations were ranked as useful by 49% (42 of 85) of respondents and as a precious tool by 42% (n = 36). Overall, participants positively rated both methods, with a median score of 7 (IQR: 5–8) for both telephone and videoconferences on a scale of 0 (no interest) to 10 (major interest).

A quarter (49 of 197) of participants maintained hospital day care for the following reasons: capsaicin patch application (39%; n = 19); intrathecal pump management (29%; n = 14); ketamine infusion (10%; n = 5); patients with complex pain, cancer or neuropathy (6%; n = 3); patients with intravenous morphine pump (2%; n = 1); and patients without any risk factors who needed treatment (2%; n = 1). In one CPS, hospital day care was maintained for all patients. 12 (25%) participants did not specify their reasons for maintaining hospital day care.

### Impact on CPS team & CPS daily work

At the time of the survey, the respondents' CPS teams were restored completely in 62% (116 of 188) of cases and partially in 35% (66 of 188) and not restored at all in 3% (six of 188). Partial return to the fully equipped CPS team was because of absence of psychologists (95%), physicians (92%), nurses (86%), secretaries and others (71%). Almost half (46%; 87 of 188) of the participants indicated that the pandemic will deeply change the way they perform their daily work. A large majority will continue to use teleconsultation when possible, in particular for at-risk patients or patients living far from the pain structure (47%; n = 41). Moreover, a majority of participants indicated that following the crisis the organization of consultation will be adapted, with an impact on the welcoming of patients and an increase in barrier measures and time between patients as a result of disinfection of the room and equipment (15%; n = 13). A few participants indicated that they had accumulated a significant backlog that would be difficult to catch up with (6%; n = 5) and that they will update the way they deal with new patients by making them fill in an auto-evaluation form before the first appointment (2%; n = 2), develop therapeutic education for patients (2%; n = 2) and have a decreased amount of time to dedicate to pain structures (2%; n = 2). Other answers included the use of computer-based files for patients instead of paper documents; increased use of hypnosis; more listening skills for caretakers in response to the high anxiety level of patients following the crisis; more reactivity, anticipation and synergy; and a change in hospitalization mode. 18 (21%) participants did not specify their treatment adaptation.

### Perceived impact on patients

According to the opinions of respondents (n = 197), disruption in the established workflow of CPSs due to the SARS-CoV-2 outbreak will have a strong impact on patients, with a median score of 8 (IQR: 7–9) on a scale of 0 (no impact) to 10 (major impact). This is related to the estimated delay in appointments before and since the crisis, which was reported to be increased from a median of 3 (IQR: 1–5) to 3.5 (IQR: 1–6) months (p < 0.001).

### Supply issues & treatment adaptation

The altered supply was noticed for medications (25%; 46 of 185) and medical devices/materials (23%; 43 of 185). Participants mostly listed midazolam (57%; n = 26), propofol (20%; n = 9), ketamine (9%; n = 4), opioids (9%; n = 4) and dronabinol (7%; n = 3). Provision issues for materials and medical devices were associated with lack of protective equipment (masks, gowns, antiseptic gel, gloves) (65%; n = 28) and TENS units (12%; n = 5). Five (11%) respondents did not specify any provision problems.

A third (33%; 61 of 185) of respondents reported that they had to change the treatment of their patients during the crisis. The majority of treatment adaptations (36%; n = 22) were associated with a decrease in or complete withdrawal of NSAID treatment. Other adaptations were associated with procedures usually administered in hospital day care (TENS, capsaicin patch, intravenous infusions) (10%; n = 6). Respondents reported an increase in anxiolytic/antidepressant prescriptions (7%; n = 4) and replacement of midazolam with other sedatives in palliative care (8%; n = 5). Other changes included the use of pharmacological treatment to replace nonpharmacological techniques unavailable during the crisis (5%; n = 3), opioid rotation (7%; n = 4), treatment adaptations linked to antiemetic supply issues (5%; n = 3) and treatment for migraine crisis (2%; n = 1). 13 (27%) participants did not specify any treatment adaptation.

## Discussion

During the COVID-19 sanitary crisis, 42% of human resources (mostly physicians and nurses) across France were detached from CPSs following the ‘Plan Blanc' deployment. A total of 47% of reallocated respondents were involved in the psychological support of caregivers, patients and families or palliative care. Such reallocation led to a more than two-thirds reduction in in-person appointments, although an effort was made to maintain physical consultations for urgent chronic pain care, cancer and palliative patients and for interventional procedures. Only a quarter of day care hospitalizations were maintained, favoring patients with the need for interventional pain treatment. As a response to this situation, telemedicine solutions were developed, with telephone calls being the most widely used method (92%). Half of respondents used videoconferences – in one-third of cases for new patients. Telemedicine solutions were ranked as important tools, with more than 40% of respondents rating them as precious. Caregivers perceived the impact of the crisis on the experience of patients to be high (eight out of ten), with an expected significant increase in access-to-care delays. One-third of responding physicians have modified patient treatment, mostly due to temporary NSAID withdrawal. A shortage in supply was reported by a quarter of respondents, mostly with regard to midazolam, anesthesia-related drugs, protective equipment and TENS units.

This clearly demonstrates that French CPSs were strongly affected by the COVID-19 crisis. Despite this serious impact, many efforts were made to maintain follow-up and new patient management. This included the preservation of in-person consultations and day care hospitalizations as well as communication with patients via the widespread use of telemedicine. No shortage of critically important medications (e.g., acetaminophen, chronic pain-specific psychoactive drugs and opioids) was reported. After the termination of ‘Plan Blanc,' all CPSs regained their staff quickly.

A few reports have begun to be published in the literature and are in line with what the authors observed in French CPSs. This includes Europe, where headache management and chronic pain centers reported huge limitations in in-person consultations, cancelled or postponed treatments and increased use of telemedicine, making access to medical treatment and patient education more difficult [6–8]. The same has also been observed in India [9], China [4], Canada [5] and the USA, where chronic pain patients had decreased access to usual care during the pandemic [6]. Some recent studies have shown that the COVID crisis exacerbated symptoms in patients suffering from chronic pain, migraine or small fiber neuropathy, with associated increased anxiety levels and a catastrophizing attitude toward pain [8–12]. The role of SARS-CoV-2 infection as a trigger for chronic pain conditions is being studied [19,20]. In line with the authors' results, pain physicians have reported increased prescribing of pain medications, particularly opioids and cannabinoids, and increased psychological distress in patients [5,6]. However, several aspects of CPS functioning and management during the ‘Plan Blanc' have to be discussed.

First, pain physicians and nurses were mostly reallocated to COVID units, with a concomitant increase in workload and night shifts. Of the 46% who were not volunteered for reallocation, almost one-third (28%) had to join different structures, including COVID units. Such mandatory reallocation, which is associated with stress, lack of equipment and high workload, may be a source of exhaustion [25] and burnout, both of which have been documented worldwide [26]. A recent study by the American Society of Interventional Pain Physicians indeed reported that 52% of participants felt burned out at the time of their survey (7 weeks after the start of the pandemic) [27]. Psychological support and listening were instituted for caregivers, mostly as local initiatives, with the participation of CPS staff. However, a long-term strategy to support healthcare workers and prevent burnout and psychological impact will be needed in case of resurgence of the crisis [26].

Second, many efforts were made to maintain follow-up and new patient management. This included the preservation of in-person consultations and day care hospitalizations as well as communication with patients via the successful use of telemedicine. There was no difference in rankings for telephone calls or video consultations for chronic pain patients, but half of the respondents reported using video consultations. However, this might be limited by material aspects, as patients need appropriate equipment to have access to video consultations.

Telehealth technology is promising for pain care, and interest has already been demonstrated, not only for patient follow-up but also for communication-based and behavioral interventions [14]. Switzerland's experience of telemedicine solutions for chronic pain patient follow-up has revealed sufficient adherence to this technology but has also evidenced that acceptance is lower in patients with higher levels of pain and anxiety [28–30]. Although telemedicine may not be a substitute for in-person interactions [4,5,23,24], it could still be an interesting tool even beyond this crisis [30,31]. For instance, the use of smartphone-based behavioral intervention has shown better adherence and is associated with greater reductions in pain and disability in adolescents with chronic pain [32].

Several participants acknowledged the importance of telemedicine, indicating a willingness to use teleconsultations in the future. This could be more convenient for some patients and may provide an effective and simple follow-up tool. Again, in the case of a sanitary crisis, a predefined telehealth strategy for maintaining follow-up as well as triage procedures to determine the necessity of an in-person appointment would be necessary.

Third, a lot of ‘fake news' circulated on social networks and in the media during the beginning of the pandemic [33]. For instance, the NSAID COVID-19 hoax phenomenon, which was immediately criticized [34], directly impacted prescription patterns in chronic pain patients. Unverified information on NSAIDs as a factor worsening the course of SARS-CoV-2 infection, published by *Le Figaro* (the oldest national newspaper), was taken seriously by many physicians [35]. Although the NSAID stop period was short, as many as 33% of pain physicians had to adapt established pain treatment in the setting of restricted access of patients to CPSs. In a crisis situation, one cannot prevent the consequences associated with the spread of nonvalidated information.

Finally, chronic pain specialists have specific knowledge related to managing care-associated pain (especially in the ICU) and guiding discussions regarding limiting or withdrawing care in end-of-life situations. According to the authors' survey, only 12% of respondents were involved in the palliative care process during the sanitary crisis. Reported international initiatives may include the use of pain physicians and nurses as palliative care specialists, not only for cancer and end-of-life patients but also in severe COVID-19 patients with ineffective critical life support [36] or in whom nursing care has been chosen (limitation of care) [37]. The CPS staff may play an important role supporting frontline colleagues and families and offering spiritual support. The skills of pain clinicians in neurocognitive interventions to reduce stress and anxiety were not optimally exploited. The latter is of particular importance for fellow caregivers dealing with an unusually high workload under conditions of restrained resources. Only CPS psychologists were massively involved in such an activity (80%). Pain management in ICU patients, which is often not seen as a priority in the context of pandemic overload, may have also been improved in collaboration with pain specialists.

The main limitation of this study is the response rate, which was difficult to evaluate because of the database structure of professional emails. However, analyzed responses came from clinicians, who were motivated to share their experience during the pandemic wave. The authors' survey reflects the staff perception of the crisis and its organizational impact but not the perception and view of the patient. Increased access-to-care delays were noted. Although statistically significant, this difference should be interpreted with caution, as it corresponds only to an estimation done by the participants and collected immediately after the end of ‘Plan Blanc'.

Recently, strategies to minimize the impact of the crisis on CPS management have been created. These include criteria for postponing interventional procedures, reducing in-person consultations (except for urgent procedures), encouraging the use of telemedicine for pre- and post-procedure evaluations, encouraging multidisciplinary therapy for the psychosocial aspects of chronic pain and avoiding new implantable devices in the context of a sanitary crisis [38–41].

## Conclusion

During the March–April 2020 COVID-19 crisis, all French CPSs were severely impacted, with a 42% reduction in human resources. Despite a two-thirds reduction in in-person appointments, the follow-up of chronic pain patients was maintained, and access to physical appointments was reserved for urgent reasons such as severe pain as well as cancer and palliative care patients. Few cases reported a disruption of pain treatment due to altered provision of drugs. Chronic pain specialists' specific knowledge of palliative/end-of-life care, neurocognitive interventions, alternative medicine and acute pain management was underutilized, and in almost 30% of specialists, who were reallocated against their wishes, burnout may be expected.

National recommendations and an action plan are necessary for efficient management of CPS centers and the use of specific knowledge of CPS human resources in a sanitary crisis. This should take into account the possible decrease in human resources and detail recommendations on the use of teleconsultations, triage procedures and criteria to maintain in-person consultations and interventional treatments as well as treatment replacement strategies for all chronic pain patients.

**Summary points**

- The first coronavirus disease 2019 wave severely impacted all specialized care structures, including chronic pain structures.
- In France, this has led to >40% reduction in human resources and two-thirds reduction in in-person appointments for patients.
- Pain specialists perceive the impact of such disruption on patient care as major.
- Of reallocated pain specialists, 30% had their occupation changed against their wishes, which is a source of potential burnout.
- The crisis promoted telemedicine consultations.
- Pain specialists' specific knowledge of palliative care, end-of-life decisions and counseling was underutilized.
- Recommendations and an action plan are necessary for more efficient management of chronic pain structure human resources.
