# Supplementary material for: Impact of COVID-19 on chronic pain structures: data from French national survey
Source: J Comp Eff Res. 2022 May 5;11(9):649–58. doi: 10.2217/cer-2022-0003 (PMC9149779; doi:10.2217/cer-2022-0003)
Supplement: Supplementary file 1 [file cer-11-649-s1.docx]

**E-appendix** 

**Supplementary Table 1:** Comparing cancer types of focus among the 15 example studies.

| *Type of Cancer* | *Number of studies using primary data from trials* | *Number of studies using EHR data* | *Number of studies using aggregate data* |
| --- | --- | --- | --- |
| Glioblastoma | 0 | 0 | 1 |
| Biliary tract cancer | 0 | 0 | 1 |
| Non-small cell lung cancer | 2 | 3 | 1 |
| Multiple myeloma | 0 | 1 | 0 |
| GE junction cancer | 0 | 1 | 0 |
| Acute lymphoblastic leukemia | 1 | 0 | 0 |
| Chronic myeloid leukemia | 1 | 0 | 0 |
| Renal cell carcinoma | 1 | 0 | 0 |
| Breast cancer | 0 | 0 | 1 |
| Small cell lung cancer | 0 | 0 | 1 |
